# The incidence of severe urinary tract infection increases after hip fracture in the elderly: a nationwide cohort study

**DOI:** 10.1038/s41598-021-83091-6

**Published:** 2021-02-09

**Authors:** Yi-Ching Lin, Ya-Chu Hsu, Wen-Tien Wu, Ru-Ping Lee, Jen-Hung Wang, Hao-Wen Chen, Ing-Ho Chen, Tzai-Chiu Yu, Cheng-Huan Peng, Kuan-Lin Liu, Chung-Yi Hsu, Kuang-Ting Yeh

**Affiliations:** 1 School of Medicine, Tzu Chi University, Hualien, 97004 Taiwan; 2 Department of Orthopedics, Hualien Tzu Chi Hospital, Buddhist Tzu Chi Medical Foundation, No. 707, Sec. 3, Zhongyang Rd., Hualien, 97002 Taiwan; 3 Institute of Medical Sciences, Tzu Chi University, Hualien, 97004 Taiwan; 4 Department of Medical Research, Hualien Tzu Chi Hospital, Buddhist Tzu Chi Medical Foundation, Hualien, 97002 Taiwan; 5 Graduate Institute of Clinical Medical Science, China Medical University, Taichung, 406040 Taiwan

**Keywords:** Trauma, Musculoskeletal system, Epidemiology, Comorbidities, Disability, Urological manifestations, Risk factors

## Abstract

Although urinary tract infection (UTI) is a common perioperative complication among elderly patients with hip fracture, its incidence and effects are often underestimated. This study investigated the effects of severe UTI (S-UTI) on elderly patients with hip fracture and the risk factors for this condition. In this retrospective nationwide cohort study, we searched Taiwan’s National Health Insurance Research Database from 2000 to 2012 for data on patients aged ≥ 50 years with hip fracture who underwent open reduction and internal fixation or hemiarthroplasty for comparison with healthy controls (i.e. individuals without hip fracture). The study and comparison cohorts were matched for age, sex, and index year at a 1:4 ratio. The incidence and hazard ratios of age, sex, and multiple comorbidities associated with S-UTI were calculated using Cox proportional hazard regression models. Among the 5774 and 23,096 patients in the study and comparison cohorts, the overall incidence of S-UTI per 100 person-years was 8.5 and 5.3, respectively. The risk of S-UTI was cumulative over time and higher in the study cohort than in the comparison cohort, particularly in those who were older, were female, or had comorbidities of cerebrovascular accident or chronic renal failure.

## Introduction

As a society ages, the prevalence of osteoporotic fractures, such as hip fracture, increases. Hip fracture can considerably impair late-life function and substantially increase the medical burden. Prompt surgery and aggressive rehabilitation may increase the survival rate and improve the overall functioning of patients with hip fracture. The complication rates of treatment on admission range from 7 to 40%^1,2^. Immediate full weight-bearing mobilisation is targeted to prevent additional harm, such as pressure sores or pneumonia. However, pre-existing comorbidities and unsteady gait often make mobilisation difficult^3^.

A prospective study reported that 1 year after hip fracture, more than 20% of patients who were previously healthy required long-term care, and approximately 80% of them relied on a walking aid to perform daily life activities^4^. Even with timely surgery and aggressive management through rehabilitation, hip fracture is associated with a high mortality rate. This mortality rate can mostly be attributed to the high prevalence of prefracture comorbidities and the high rate of postoperative complications, such as surgical wound infection, pneumonia, bleeding, ileus, delirium, and urinary tract infection (UTI)^5,6^, which is a highly common postoperative complication in numerous surgical fields^7^. The rate of UTI after surgery for hip fracture may be underestimated and associated with inferior functional outcomes^8^. UTI does not seem to affect the rate of perioperative wound infection or in-hospital mortality. However, severe UTI (S-UTI) often requires further intervention. S-UTI is defined as symptomatic UTI requiring hospitalisation to prevent severe adverse sequelae, including death. Studies on UTI during and after recovery from hip fracture have seldom been performed. This nationwide population-based cohort study investigated the incidence of and risk factors for S-UTI after hip fracture surgery. The present findings can serve as a reference for the provision of special care to reduce the risk of postoperative S-UTI, thereby improving the quality of life of affected patients and reducing medical expenditure.

## Results

**Figure 1:**
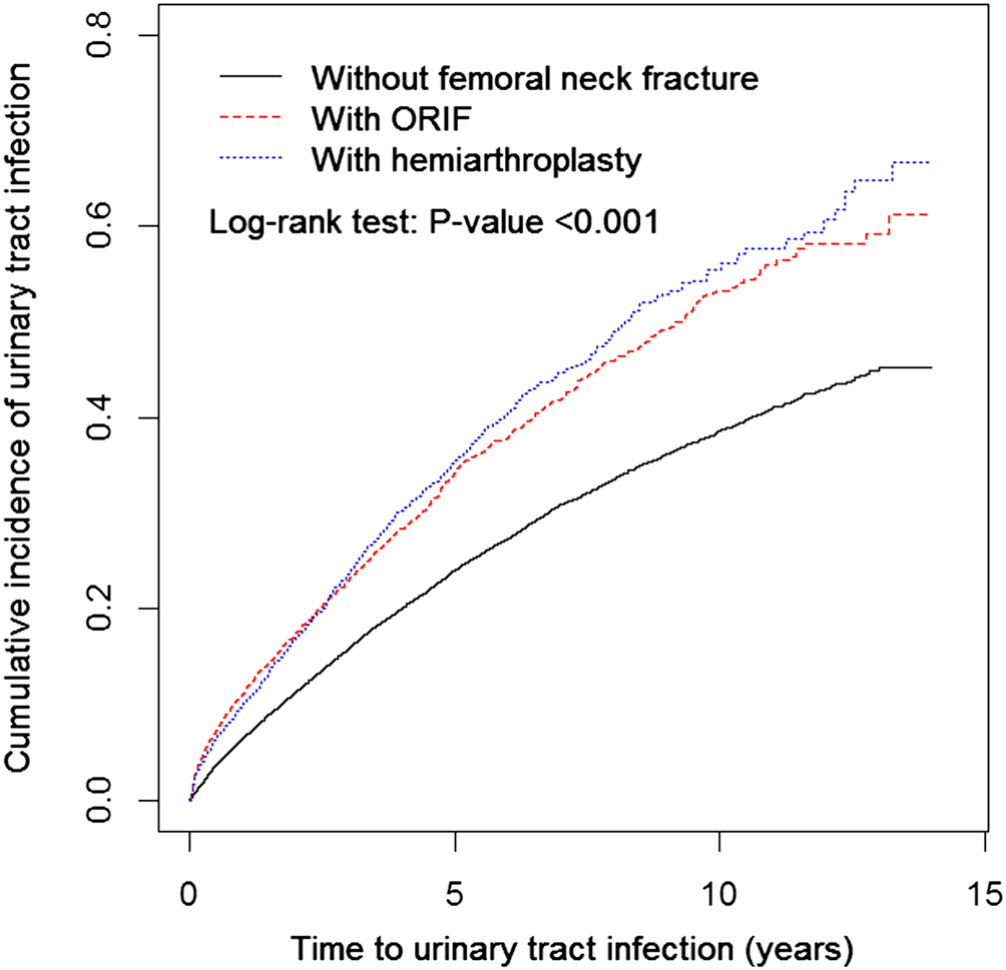
Cumulative incidences of urinary tract infection for ORIF, hemiarthroplasty and non-femoral neck fracture cohorts.

The study and comparison cohorts comprised 5774 and 23,096 patients, respectively. The study cohort was further divided by method of hip fracture surgery. The open reduction and internal fixation (ORIF) and hemiarthroplasty groups contained 3469 (60.1%) and 2305 (39.9%) patients, respectively. The comparison cohort comprised patients from the same database without hip fracture during the same period. Patients with hip fracture who did not undergo surgery were excluded because they comprised less than 1% of patients with hip fracture and surgery is the primary intervention for this type of injury. No significant between-cohort differences were observed in terms of age; sex; follow-up period; or comorbidities, including hypertension (HTN), diabetes mellitus (DM), hyperlipidaemia, depression, coronary artery disease (CAD), cardiovascular accident (CVA), and chronic renal failure (CRF; Table 1). In both cohorts, patients with any history of S-UTI, those with a confirmed diagnosis of hospital-acquired UTI from prolonged Foley catheter placement, and those receiving antibiotics more than 24 h postoperatively before and during the admission period for hip fracture surgery were excluded. The cumulative incidence of S-UTI was significantly higher in the ORIF and hemiarthroplasty groups than in the control group, according to a Kaplan–Meier analysis (log-rank test, *P* < 0.001; Fig. 1). The overall incidence ratios of S-UTI for the control, ORIF, and hemiarthroplasty groups per 100 person-years were 5.3, 8.3, and 8.7, respectively (Table 2). The ORIF and hemiarthroplasty groups exhibited an increased risk of S-UTI compared with the control group, even after adjustment for sex; age; and comorbidities of HTN, DM, CAD, hyperlipidaemia, CRF, CVA, and depression (adjusted hazard ratios [aHRs] = 1.65 and 1.55, 95% confidence interval [CI] 1.55, 1.77 and 1.44, 1.68, respectively). No significant differences were noted between the ORIF and hemiarthroplasty groups (aHR = 0.93, 95% CI 0.85, 1.03; Table 2). The increased incidence of S-UTI in the ORIF group was observed across all strata. The same phenomenon was noted in the hemiarthroplasty group across all strata, except for the stratification by CRF (Table 2). Comorbidity analysis revealed that CRF and CVA were significantly associated with S-UTI incidence (*P* = 0.0097 and 0.0038, respectively; Table 3). The aHR of S-UTI in men was 0.77 times higher than that in women (95% CI 0.74, 0.81). Compared with that in patients aged 50–59 years, the S-UTI risk was 1.87, 3.42, and 5.57 times higher in patients aged 60–69 years (95% CI 1.58, 2.22), 70–79 years (95% CI 2.93, 4.01), and > 80 years (95% CI 4.76, 6.52; Table 3), respectively. HTN, DM, CAD, hyperlipidaemia, and depression were all significantly associated with S-UTI incidence (*P* < 0.05; Table 3). The aHR for S-UTI in the patients with CRF was 4.85 times higher than that in those without CRF (95% CI 4.45, 5.29). In the patients with CVA, it was 1.56 times higher than that in those without CVA (95% CI 1.48, 1.64; Table 3). The aHRs for S-UTI interacting with CRF and CVA were 5.05 (95% CI 4.58, 5.56) and 1.85 (95% CI 1.50, 1.68) in the control group, 7.77 (95% CI 6.06, 9.97) and 2.55 (95% CI 2.31, 2.81) in the ORIF group, and 5.85 (95% CI 4.41, 7.76) and 2.32 (95% CI 2.07, 2.60; Table 4) in the hemiarthroplasty group, respectively.

**Table 1:** Baseline characteristics in study cohorts with hip fx undergoing surgery and without hip fx.

	Hip fx				Standardized mean difference
	No (n = 23,096)		Yes (n = 5774)		
	n	%	n	%	
**Surgery type**					
ORIF			3469	60.1	
Hemiarthroplasty			2305	39.9	
**Sex**					0.05
Female	12,514	54.2	3182	55.1	
Male	10,582	45.8	2592	44.9	
**Age (years)**					0.02
50–59	1900	8.2	472	8.2	
60–69	3550	15.4	869	15.1	
70–79	8540	37	2165	37.5	
80+	9106	39.4	2268	39.3	
Mean (SD)	76.3	9.94	76.2	9.84	
**Comorbidity**					
HTN	16,942	73.4	4205	72.8	0.05
DM	9148	39.6	2281	39.5	0.02
Hyperlipidemia	7245	31.4	1802	31.2	0.01
CRF	457	2	113	2	0.01
Depression	2710	11.7	712	12.3	0.02
CAD	10,803	46.8	2678	46.4	0.05
CVA	8792	38.1	2193	38	0.01
Mean of follow-up period of S-UTI (Mean/SD) (years)	4.14	3.19	3.74	3.19	0.09

A standardized mean difference ≤ 0.10 indicates a negligible difference between the two cohorts.

*CAD* coronary artery disease, *CRF* chronic renal failure, *CVA* cerebrovascular accident, *DM* diabetes mellitus, *Fx* fracture, *HTN* hypertension, *ORIF* open reduction and internal fixation, *S-UTI* severe urinary tract infection.

**Table 2:** Incidences and hazard ratios of S-UTI for ORIF, hemiarthroplasty, and non-hip fx cohorts.

Code	Non-hip fx			ORIF			Hemiarthroplasty			Hazard ratio (95% confidence interval)						
	0			1			2			Crude			Adjusted			*P* for interaction
	Event	PY	IR	Event	PY	IR	Event	PY	IR	1 vs 0	2 vs 0	2 vs 1	1 vs 0	2 vs 0	2 vs 1	
**Overall**	5106	95,710	5.3	1071	12,927	8.3	756	8685	8.7	1.54 (1.45, 1.65)***	1.62 (1.5, 1.75)***	1.04 (0.95–1.15)	1.65 (1.55, 1.77)***	1.55 (1.44, 1.68)***	0.93 (0.85–1.03)	
**Sex**																
Female	3139	52,931	5.9	629	6827	9.2	504	5634	8.9	1.55 (1.42, 1.68)***	1.5 (1.37, 1.65)***	0.97 (0.86–1.09)	1.51 (1.38, 1.64)***	1.39 (1.27, 1.53)***	0.92 (0.82–1.03)	
Male	1967	42,779	4.6	442	6100	7.2	252	3051	8.3	1.57 (1.41, 1.74)***	1.78 (1.56, 2.02)***	1.13 (0.97–1.32)	1.9 (1.72, 2.11)***	1.97 (1.73, 2.25)***	1.03 (0.88–1.21)	
**Age (years)**																
50–59	119	10,977	1.1	40	2164	1.8	12	315	3.8	1.69 (1.18, 2.42)**	3.48 (1.92, 6.3)***	2.05 (1.07–3.91)*	1.72 (1.19, 2.47)**	2.3 (1.23, 4.3)**	1.33 (0.68–2.62)	
60–69	485	18,865	2.6	101	2276	4.4	99	1957	5.1	1.72 (1.39, 2.13)***	1.96 (1.58, 2.44)***	1.14 (0.86–1.50)	1.7 (1.37, 2.11)***	1.76 (1.42, 2.19)***	1.03 (0.78–1.36)	
70–79	2002	37,630	5.3	430	4595	9.4	325	3917	8.3	1.75 (1.58, 1.94)***	1.55 (1.38, 1.75)***	0.88 (0.76–1.02)	1.78 (1.6, 1.97)***	1.6 (1.42, 1.8)***	0.90 (0.78–1.04)	
80 +	2500	28,237	8.9	500	3891	13	320	2496	13	1.45 (1.31, 1.59)***	1.44 (1.28, 1.62)***	0.99 (0.86–1.14)	1.5 (1.36, 1.65)***	1.39 (1.24, 1.56)***	0.92 (0.80–1.06)	
**Comorbidity**																
HTN																
No	1038	29,247	3.5	228	4548	5	142	2488	5.7	1.41 (1.22, 1.63)***	1.6 (1.35, 1.91)***	1.13 (0.92–1.40)	1.53 (1.32, 1.76)***	1.6 (1.34, 1.91)***	1.05 (0.85–1.29)	
Yes	4068	66,462	6.1	843	8379	10	614	6197	9.9	1.63 (1.51, 1.76)***	1.61 (1.48, 1.75)***	0.98 (0.88–1.09)	1.69 (1.57, 1.82)***	1.55 (1.42, 1.69)***	0.91 (0.82–1.01)	0.210
DM																
No	2731	60,377	4.5	603	8372	7.2	407	5526	7.4	1.59 (1.45, 1.73)***	1.62 (1.46, 1.8)***	1.02 (0.90–1.15)	1.66 (1.52, 1.82)***	1.5 (1.35, 1.66)***	0.90 (0.79–1.02)	
Yes	2375	35,332	6.7	468	4555	10	349	3159	11	1.51 (1.37, 1.67)***	1.63 (1.46, 1.82)***	1.07 (0.93–1.23)	1.62 (1.47, 1.79)***	1.61 (1.44, 1.8)***	0.99 (0.86–1.13)	0.764
Hyperlipidemia																
No	3627	66,219	5.5	770	9295	8.3	510	5912	8.6	1.51 (1.39, 1.63)***	1.57 (1.43, 1.72)***	1.04 (0.93–1.16)	1.61 (1.49, 1.74)***	1.52 (1.38, 1.66)***	0.94 (0.84–1.05)	
Yes	1479	29,491	5	301	3632	8.3	246	2773	8.9	1.65 (1.45, 1.86)***	1.75 (1.53, 2.01)***	1.06 (0.90–1.26)	1.74 (1.54, 1.97)***	1.65 (1.44, 1.89)***	0.94 (0.79–1.11)	0.291
CRF																
No	4650	94,193	4.9	1008	12,776	7.9	707	8532	8.3	1.59 (1.49, 1.7)***	1.67 (1.54, 1.8)***	1.04 (0.95–1.15)	1.66 (1.55, 1.78)***	1.58 (1.46, 1.72)***	0.95 (0.86–1.05)	
Yes	456	1517	30	63	151	42	49	153	32	1.45 (1.11, 1.89)**	1.11 (0.82, 1.49)	0.76 (0.52–1.10)	1.56 (1.19, 2.05)**	1.2 (0.88, 1.63)	0.76 (0.52–1.12)	0.0097**
Depression																
No	4454	85,443	5.2	943	11,583	8.1	654	7635	8.6	1.55 (1.45, 1.67)***	1.63 (1.5, 1.77)***	1.05 (0.95–1.16)	1.67 (1.56, 1.79)***	1.56 (1.44, 1.69)***	0.93 (0.55–0.64)	
Yes	652	10,267	6.4	128	1344	9.5	102	1049	9.7	1.49 (1.24, 1.81)***	1.52 (1.23, 1.87)***	1.01 (0.78–1.31)	1.53 (1.27, 1.85)***	1.51 (1.22, 1.86)***	0.98 (0.75–1.27)	0.7879
CAD																
No	2342	54,039	4.3	534	7722	6.9	370	4781	7.7	1.59 (1.45, 1.75)***	1.78 (1.59, 1.98)***	1.11 (0.97–1.27)	1.74 (1.58, 1.91)***	1.6 (1.43, 1.79)***	0.91 (0.80–1.05)	
Yes	2764	41,671	6.6	537	5205	10	386	3904	9.9	1.54 (1.41, 1.69)***	1.48 (1.33, 1.65)***	0.96 (0.84–1.09)	1.57 (1.43, 1.72)***	1.51 (1.35, 1.68)***	0.96 (0.84–1.09)	0.0648
CVA																
No	2608	64,539	4	597	9000	6.6	405	5554	7.3	1.64 (1.5, 1.79)***	1.8 (1.62, 1.99)***	1.09 (0.96–1.24)	1.7 (1.55, 1.86)***	1.63 (1.47, 1.82)***	0.96 (0.84–1.09)	
Yes	2498	31,170	8	474	3927	12	351	3130	11	1.49 (1.35, 1.65)***	1.39 (1.25, 1.56)***	0.93 (0.81–1.07)	1.58 (1.44, 1.75)***	1.46 (1.31, 1.63)***	0.92 (0.80–1.05)	0.0038**

Adjusted hazard ratio for controlling for sex, age, and every comorbidity in Table 3.

*HR* hazard ratio, *IR* incidence rate, per 100 person-years, *CAD* coronary artery disease, *CI* confidence interval, *CRF* chronic renal failure, *CVA* cerebrovascular accident, *DM* diabetes mellitus, *fx* fracture, *HTN* hypertension, *ORIF* open reduction and internal fixation, *PY* person-years, *S-UTI* severe urinary tract infection.

*P < 0.05, **P < 0.01, ***P < 0.001.

**Table 3:** Cox model measured hazard ratio and 95% confidence intervals of S-UTI associated with ORIF, hemiarthroplasty, non-hip fx cohorts and covariates.

Characteristics	S-UTI event no. (n = 6933)	Crude			Adjusted		
		HR	(95% CI)	*P* value	HR	(95% CI)	*P* value
**Hip fx**							
No	5106	1	Reference		1	Reference	
ORIF	1071	1.54	(1.45, 1.65)	< 0.001	1.65	(1.55, 1.77)	< 0.001
Hemiarthroplasty	756	1.62	(1.5, 1.75)	< 0.001	1.55	(1.44, 1.68)	< 0.001
**Sex**							
Female	4272	1	Reference		1	Reference	
Male	2661	0.78	(0.74, 0.82)	< 0.001	0.77	(0.74, 0.81)	< 0.001
**Age (years)**							
50–59	171	1	Reference		1	Reference	
60–69	685	2.32	(1.97, 2.75)	< 0.001	1.87	(1.58, 2.22)	< 0.001
70–79	2757	4.64	(3.98, 5.42)	< 0.001	3.42	(2.93, 4.01)	< 0.001
80 +	3320	7.3	(6.26, 8.52)	< 0.001	5.57	(4.76, 6.52)	< 0.001
**Comorbidity**							
HTN							
No	1408	1	Reference		1	Reference	
Yes	5525	1.71	(1.61, 1.82)	< 0.001	1.2	(1.12, 1.28)	< 0.001
DM							
No	3741	1	Reference		1	Reference	
Yes	3192	1.45	(1.38, 1.52)	< 0.001	1.46	(1.38, 1.53)	< .001
Hyperlipidemia							
No	4907	1	Reference		1	Reference	
Yes	2026	0.92	(0.88, 0.97)	0.002	0.74	(0.7, 0.78)	< 0.001
CRF							
No	6365	1	Reference		1	Reference	
Yes	568	5.51	(5.06, 6)	< 0.001	4.85	(4.45, 5.29)	< 0.001
Depression							
No	6051	1	Reference		1	Reference	
Yes	882	1.18	(1.1, 1.27)	< 0.001	1.1	(1.02, 1.18)	0.01
CAD							
No	3246	1	Reference		1	Reference	
Yes	3687	1.46	(1.4, 1.53)	< 0.001	1.06	(1.01, 1.12)	0.02
CVA							
No	3610	1	Reference		1	Reference	
Yes	3323	1.86	(1.77, 1.95)	< 0.001	1.56	(1.48, 1.64)	< 0.001

Adjusted HR: adjusted for femoral neck fracture, sex, age, and every comorbidity in Table 2 in Cox proportional hazards regression.

*CAD* coronary artery disease, *CI* confidence interval, *CRF* chronic renal failure, *CVA* cerebrovascular accident, *DM* diabetes mellitus, *fx* fracture, *HR* hazard ratio, *HTN* hypertension, *ORIF* open reduction and internal fixation, *S-UTI* severe urinary tract infection.

**Table 4:** Cox proportional hazard regression analysis for the risk of hip fx-associated S-UTI with interaction of CRF and CVA.

Variables	*n*	Event no	Adjusted HR (95% CI)	*P* value^a^
**Hip fx status**				
CRF				0.010*
No				
No	22,639	4650	1 (Reference)	
Yes	457	456	5.05 (4.58–5.56)	< 0.0001
ORIF				
No	3405	1008	1.66 (1.55–1.78)	< 0.0001
Yes	64	63	7.77 (6.06–9.97)	< 0.0001
Hemiarthroplasty				
No	2256	707	1.59 (1.46–1.72)	< 0.0001
Yes	49	49	5.85 (4.41–7.76)	< 0.0001
**Hip fx status**				
CVA				0.004*
No				
No	14,304	2608	1 (Reference)	
Yes	8792	2498	1.58 (1.50–1.68)	< 0.0001
ORIF				
No	2208	597	1.69 (1.54–1.85)	< 0.0001
Yes	1261	474	2.55 (2.31–2.81)	< 0.0001
Hemiarthroplasty				
No	1373	405	1.64 (1.47–1.82)	< 0.0001
Yes	932	351	2.32 (2.07–2.60)	< 0.0001

Adjusted HR: adjusted for femoral neck fracture, sex, age, and the comorbidities in Cox proportional hazards regression.

*HR* hazard ratio, *CI* confidence interval, *CRF* chronic renal failure, *CVA* cerebrovascular accident, *fx* fracture, *ORIF* open reduction and internal fixation, *S-UTI* severe urinary tract infection.

^a^*P* value for interaction.

## Discussion

This study discovered an increased risk of S-UTI after surgery for hip fracture compared with the risk in individuals without hip fracture. UTIs, which are among the most common bacterial infections in older patients with hip fracture^9^, can be symptomatic or asymptomatic^10^. The present study examined the postoperative incidence of and risk factors for S-UTI to prevent its occurrence during recovery from hip fracture. The incidence of S-UTI was significantly higher in the study cohort than in the comparison cohort and increased over time. Older and female patients were even more likely to develop S-UTI. A 2005 study reported significant differences of 16% and 4% in UTI occurrence after hip fracture surgery between patients older and younger than 65 years, respectively^11^. Age is a crucial factor that contributes to patients’ overall health, prefracture mobility, and postoperative recovery rate^12^. The primary causes of UTI include postoperative urinary retention and neurogenic bladder dysfunction^13^. UTIs are more common in women than in men because their urethras are relatively short and close to the anus and vagina. *Escherichia coli* is the most common pathogen implicated in UTIs^14^. These findings are consistent with those of other studies^15–17^.

Logistic regression analysis revealed that the comorbidities of HTN, DM, CAD, hyperlipidaemia, CRF, CVA, and depression were all significantly correlated with high S-UTI incidence. CRF and CVA were the most critical effect modifiers. CRF is associated with UTI for two main reasons. First, oliguria in CRF may cause stagnation along the urinary tract, thereby promoting bacterial growth. Second, defective urinary concentration affects the concentration of antibacterial substances in the urine^18,19^. In addition, acute urine retention and other micturition disorders, which occur frequently in elderly patients after hip fracture surgery, may aggravate UTIs to develop into S-UTIs^13^. UTIs affect between 10 and 19% of patients with CVA and are highly common poststroke. Comorbid CVA in elderly patients with hip fracture may increase the incidence of S-UTI^20^. According to Donegan et al., classes III and IV in the American Society of Anaesthesiologists classification of physiological status are strongly associated with perioperative complications, such as CVA and CRF, in elderly patients after hip fracture surgery^21^. This result is consistent with the present findings.

We divided the study group into ORIF and hemiarthroplasty groups for analysis in the present study. Each surgical method was indicated for hip fracture on the basis of fracture type and classification. According to a previous literature review, hemiarthroplasty is more cost effective than ORIF with screw fixation for nondisplaced femoral neck fractures in elderly patients with low function demand^22^. Another study revealed no differences in 30-day mortality rates but significant differences in respiratory complications among ORIF, hemiarthroplasty, and total hip arthroplasty groups^23^. The present study results reveal no significant difference in S-UTI incidence between the ORIF and hemiarthroplasty groups. Patient characteristics and comorbidities may be as crucial as fracture type is for the selection of an appropriate surgical method. We also excluded patients who underwent surgery more than 24 h after fracture because a previous meta-analysis of more than 190,000 patients indicated that delayed surgery for hip fracture is associated with a significantly increased risk of death and pressure sores^24^. In 2004, the Journal of the American Medical Association revealed that surgery within 24 h of fracture is associated with reduced pain and duration of hospitalisation; therefore, patients with hip fracture in stable medical condition should undergo surgery as soon as possible^25^.

This study did not include patients who did not undergo surgery for hip fracture because surgery is the primary intervention for this type of injury^25^. The large sample size was a strength of this study; the comprehensive coverage of the National Health Insurance (NHI) system (covering > 95% of the Taiwanese population) may have minimised selection and nonresponse biases. However, this study had some limitations. First, data on lifestyle factors, detailed disease symptoms, personal characteristics, and biochemical indices, which may be influential sources of bias, were unavailable because the National Health Insurance Research Database (NHIRD) does not provide this information. Second, because our results are based on data from Taiwan, the present findings may not be directly generalisable to Caucasian or African populations. Despite these limitations, the present study indicated the importance of preventing S-UTI after surgical intervention for hip fracture in elderly patients. Clinicians and family members alike should collaborate to prevent S-UTI and thus improve the quality of life of patients during and after recovery from hip fracture.

## Conclusions

The S-UTI incidence in the study cohort was higher than that in the comparison cohort, particularly in patients who were older, female, or had comorbid CVA or CRF. To increase patients’ postoperative quality of life, further prevention or protection protocols for these patients at high risk of S-UTI should be enacted earlier during the recovery period after hip fracture.

## Methods

The main data source for this study was the NHIRD, access to which is provided by the National Health Research Institute. The NHIRD is a nationwide database covering approximately 99% of the 23.74 million residents of Taiwan who are enrolled in the NHI programme, which was launched on March 1, 1995. We used scrambled identification to link three additional data sources, namely the Registry for Catastrophic Illness Patient Database (RCIPD), Longitudinal Health Insurance Database 2000 (LHID 2000), and Registry of Beneficiaries (RB)^26^. Records in the NHIRD correspond with the codes of the International Classification of Diseases, Ninth Revision, Clinical Modification (ICD-9-CM). This study was approved by the Ethics Review Board of China Medical University and China Medical University Hospital (CMUH-104-REC2-115), and all analyses were performed in accordance with relevant guidelines and regulations. The RCIPD, LHID 2000, and RB, as legal and delinked databases for research, are anonymised and maintained by the National Health Research Institute with confidentiality in accordance with the Personal Electronic Data Protection Law. The requirement for informed consent of this study was waived by the Ethics Review Board of China Medical University and China Medical University Hospital on the basis of the Personal Electronic Data Protection Law.

We searched the RCIPD from 1 January 2000 to 31 December 2012 for patients aged ≥ 50 years receiving surgical intervention for newly diagnosed hip fracture (ICD-9-CM codes 820.0–820.9; codes for ORIF and hemiarthroplasty: 64029B and 64170B). These patients constituted the study cohort. S-UTI was defined as UTI requiring hospitalisation (ICD-9-CM code 599.0). The comparison cohort comprised patients from the LHID 2000 without hip fracture during the same period. In both cohorts, patients with any history of S-UTI before or during the admission period for hip fracture surgery, those with a confirmed diagnosis of hospital-acquired UTI from prolonged Foley catheter placement, and those receiving antibiotics more than 24 h postoperatively before and during the admission period for hip fracture surgery were excluded. In the hip fracture cohort, patients who did not undergo surgery within 72 h after fracture were excluded. The study and comparison cohorts were frequency matched for age, sex, index year, and comorbidities at a 1:4 ratio.

All individuals were followed from the index date to hospitalisation for S-UTI until their date of death, withdrawal from the NHI programme, or 31 December 2012, whichever occurred first. Some demographic factors and comorbidities that may be associated with S-UTI were also identified. These included sexes, age, and the following comorbidities: HTN (ICD-9-CM codes 401–405), DM (ICD-9-CM code 250), hyperlipidaemia (ICD-9-CM code 272), depression (ICD-9-CM codes 296.2, 296.3, 296.82, 300.4, 309.0, 309.1, and 311), CAD (ICD-9 codes 410–414), CVA (ICD-9 codes 430–438), and CRF (ICD-9 code 585).

### Statistical analysis

The standardised mean differences in sex, age, comorbidities, and follow-up duration were subjected to further analysis. The incidence rate was defined as the number of S-UTI events divided by person-years. Crude hazard ratios, aHRs, and 95% CIs were calculated using the multivariable Cox proportional hazard regression model (adjusted for sex, age, and comorbidities). The Kaplan–Meier method was used to determine the cumulative incidence of S-UTI in patients with and without hip fracture, and the log-rank test was used to examine its significance. The analyses were performed using SAS software, Version 9.4 of the SAS System for Unix (SAS Institute, Cary, NC, USA). A *P* value of < 0.05 was considered to be significant.

### Ethics approval and consent to participate

This study was approved by the Ethics Review Board of China Medical University and China Medical University Hospital (CMUH-104-REC2-115) and confirmed that all the experiments were performed in accordance with relevant guidelines and regulations. The requirement for informed consent of this study was waived by the Ethics Review Board based on the Personal Electronic Data Protection Law because the NHIRD database are anonymized and maintained by the National Health Research Institute with confidentiality according to the law.

## Data Availability

The datasets used and analyzed during the current study are available from the corresponding author on reasonable request.
